# Chemical Composition, Cytotoxicity, and Encapsulation of Lavender Essential Oil (*Lavandula angustifolia*) in Alginate Hydrogel—Application and Therapeutic Effect on Animal Model

**DOI:** 10.3390/molecules30142931

**Published:** 2025-07-11

**Authors:** Michalina Adaszyńska-Skwirzyńska, Yu-Hsiang Yu, Paweł Konieczka, Krzysztof Kozłowski, Dorota Witkowska, Andrzej Dybus, Beata Hukowska-Szematowicz, Magdalena Jędrzejczak-Silicka, Mateusz Bucław, Artur Bartkowiak

**Affiliations:** 1 Department of Monogastric Animal Sciences, Faculty of Biotechnology and Animal Husbandry, West Pomeranian University of Technology in Szczecin, Janickiego St. 29, 71-270 Szczecin, Poland; mateusz.buclaw@zut.edu.pl; 2 Department of Biotechnology and Animal Science, National Ilan University, No. 1, Sec. 1, Shennong Rd., Yilan City 26047, Taiwan; yuyh@niu.edu.tw; 3 Department of Poultry Science and Apiculture, University of Warmia and Mazury in Olsztyn, Oczapowskiego St. 5, 10-719 Olsztyn, Poland; pawel.konieczka@uwm.edu.pl (P.K.); krzysztof.kozlowski@uwm.edu.pl (K.K.); 4 The Kielanowski Institute of Animal Physiology and Nutrition, Polish Academy of Sciences, Instytucka St. 3, 05-110 Jablonna, Poland; 5 Department of Animal Welfare and Research, Faculty of Animal Bioengineering, University of Warmia and Mazury in Olsztyn, Oczapowskiego St. 5, 10-718 Olsztyn, Poland; dorota.witkowska@uwm.edu.pl; 6 Department of Genetics, West Pomeranian University of Technology, Piastów Av. 45, 70-311 Szczecin, Poland; andrzej.dybus@zut.edu.pl; 7 Institute of Biology, University of Szczecin, Felczaka St. 3c, 71-412 Szczecin, Poland; beata.hukowska-szematowicz@usz.edu.pl; 8 Molecular Biology and Biotechnology Center, University of Szczecin, Wąska St. 13, 71-412 Szczecin, Poland; 9 Laboratory of Biostatistics, Bioinformatics and Animal Breeding, West Pomeranian University of Technology in Szczecin, Janickiego St. 29, 71-270 Szczecin, Poland; magdalena.jedrzejczak@zut.edu.pl; 10 Center of Bioimmobilisation and Innovative Packaging Materials, Faculty of Food Sciences and Fisheries, West Pomeranian University of Technology in Szczecin, Janickiego St. 35, 71-270 Szczecin, Poland; artur-bartkowiak@zut.edu.pl

**Keywords:** lavender essential oil, cytotoxicity, alginate hydrogel, biological activity, cecal microbiota, therapeutic effect

## Abstract

Lavender essential oil (LEO) was analyzed using gas chromatography coupled with a mass selective detector (GC-MS), detecting linalool and linalyl acetate as its major constituents. The biological activity of the LEO was evaluated in vitro using a normal mouse fibroblast cell line (L929), where it showed no cytotoxic effects. To assess its therapeutic effect in vivo, a broiler chicken model (Ross 308) was employed. Birds were divided into three groups: the control group (C) without any hydrogel supplementation; the H group, supplemented with alginate hydrogel capsules without LEO; and the HE groups, which received hydrogel capsules containing immobilized LEO. Capsules were provided on chick paper for voluntary intake from day 1 to day 10. At the end of the production cycle, the cecum was dissected and preserved for subsequent molecular analyses. Results demonstrated that dietary supplementation with alginate hydrogel containing immobilized LEO (HE group) positively influenced the production parameters and intestinal health in broiler chickens. Dietary supplementation with alginate hydrogel-encapsulated LEO exerts therapeutic effects in broilers.

## 1. Introduction

In recent years, there has been growing interest in developing polymer-based systems for controlled delivery of drugs or bioactive substances. While these systems offer better therapeutic properties compared with conventional forms, they are not sufficiently responsive to dynamic metabolic changes occurring in the body. Therefore, the drug delivery system must be optimized to cooperate with the body’s self-regulatory mechanisms. Hydrogel systems have emerged as particularly promising solutions due to their ability to respond to various physiological stimuli, including temperature, environmental pH, ionic strength, and the presence of specific biomolecules (e.g., antibodies, glucose, morphine, urea, and metal ions) [1,2,3]. Hydrogels consist of at least two components, i.e., a hydrophilic polymer (or biopolymer) and water. Among biopolymers, those containing chitosan, collagen, quinine, or alginates demonstrate significant application potential. Alginates are naturally occurring polysaccharides, with sodium alginate—an alginic acid salt—present in the cell walls of brown seaweeds (*Phaeophyceae)* and certain bacterial mucus secretions [4]. At the molecular level, alginate is a linear copolymer composed of two monomeric units: α-L-guluronic acid (G) and β-D-mannuronic acid (M) [3,5,6,7]. These units are epimers that differ in their spatial configuration at the C-5 carbon position, a structural feature that enables glycosidic bond formation. Alginate hydrogels can be formed through both ionic and covalent crosslinking methods [5,7]. The ionic crosslinking process involves an exchange of sodium ions for calcium ions, achieved by combining aqueous sodium alginate with a calcium chloride solution. In recent years, alginate hydrogels have gained increasing attention from both researchers and industry, particularly in the food, cosmetic, and pharmaceutical sectors [4]. The properties of alginate structures vary significantly depending on the crosslinking ions used, enabling diverse applications. However, these characteristics are influenced by multiple interdependent factors, including the polymer type used for synthesis, crosslinking density, functional groups, and the ionic strength of absorbed liquid [2,7,8]. Alginates have been used as functional biomaterials due to their bioactive properties, resulting from diverse chemical compositions and variable molecular weights. The biological activity of alginate was first demonstrated in early animal transplantation trials involving encapsulated Langerhans islets for diabetes control [9].

The high-water content of hydrogels facilitates the diffusion of various compounds both into and out of their structure, a property that has enabled their application in the design of controlled drug delivery systems. Additional characteristics of hydrogels, such as their softness and plasticity, have further supported the growing application of hydrogels in biomedical products and the pharmaceutical industry [6,7,10,11,12,13]. For years, researchers have sought safe and biocompatible materials capable of delivering growth factors, drugs, peptides, or cells. These materials must not only possess suitable mechanical properties, appropriate structure, and composition but also be readily available and cost-effective [10]. Alginates have emerged as functional biomaterials due to their favorable interactions with biological systems, resulting from the wide range of possible chemical compositions and polymer molecular weights. When administered in capsule form, alginate-based hydrogels can be engineered to deliver bioactive substances, such as essential oils (**EOs**), to specific regions of the gastrointestinal tract [10]. EOs exhibit multidirectional biological effects [14]. Animal studies have demonstrated their capacity to improve feed palatability and intake, regulate digestive functions, and modulate gastrointestinal microbiota, consequently improving growth performance and feed efficiency—a particularly relevant benefit given rising feed costs [15]. Among EOs, lavender EO (**LEO**; *Lavandula angustifolia*) deserves particular attention due to its proven antibacterial, antifungal, antioxidant, sedative, and immunostimulatory properties [16,17]. Its main chemical constituents include linalool, linalyl acetate, lavandulol, and 4-terpineol, known for their broad biological and pharmacological activities. LEO exhibits dual functionality by inhibiting pathogenic bacterial flora while stimulating the growth of beneficial microbiota [15,18,19]. Although research on the use of EOs in poultry production is steadily growing, the administration of EOs in the form of alginate hydrogel capsules has not yet been described. This novel delivery approach motivated our investigation into alginate hydrogel supplementation for broilers. Specifically, the study evaluated the chemical composition and cytotoxicity of LEO, as well as the effects of administering alginate hydrogel capsules, both with and without immobilized LEO, on production parameters and cecal microbiota in broiler chickens.

## 2. Results

### 2.1. Essential Oil

The analyzed LEO contained 29 chemical compounds, with linalool, linalyl acetate, 4-terpineol, lavandulyl acetate, β-caryophyllene, and caryophyllene oxide as dominant constituents. The chemical profile of LEO complied with all European Pharmacopoeia [20] specifications for *Lavandula angustifolia* oils. The detailed chemical composition of LEO is presented in Table 1.

**Figure 1:**
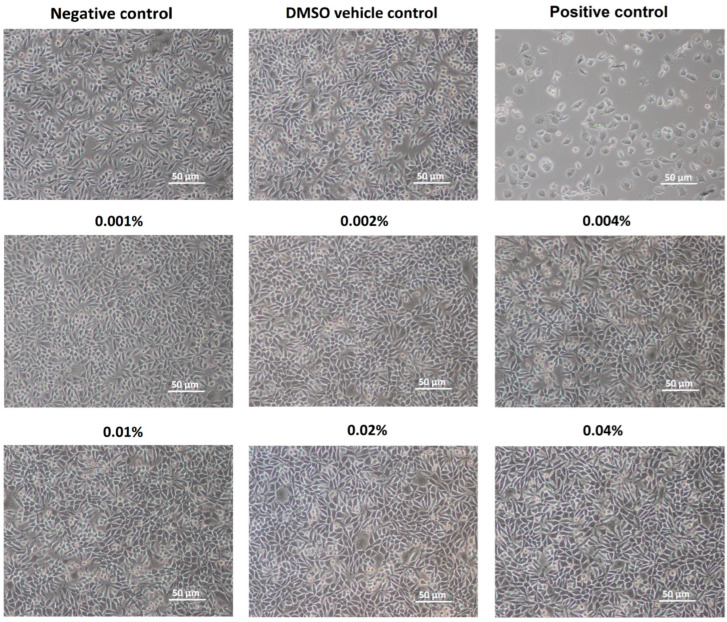
Morphology of the L929 cell line after 48 h exposure to lavender essential oil.

**Figure 2:**
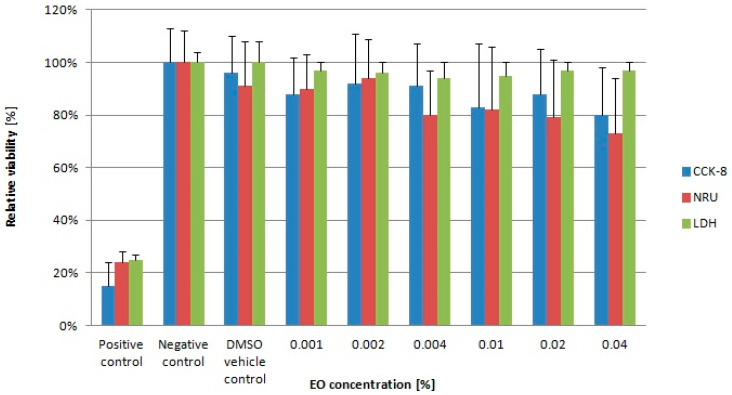
Relative viability of L929 cells after 48 h incubation with lavender essential oil (LEO).

The morphological analysis of the L929 cell line exposed to commercial LEO revealed no changes in cell morphology or number, even at the highest EO concentrations (at 0.02% and 0.04%) after 48 h incubation (Figure 1). The biological effects of LEO were further evaluated using the CCK-8, neutral red uptake (NRU), and LDH leakage assays (Figure 2). Results of the CCK-8 assay indicated minimal changes in relative cell viability, with the lowest values (83% and 80%) recorded for LEO concentrations of 0.01% and 0.04%, respectively. The NRU assays demonstrated slightly lower records in relative cell viability values across the tested concentration range (0.004% to 0.04%) compared with the CCK-8 results. The lowest viability recorded in this assay was 73% following 48 h exposure to 0.04% LEO. The LDH leakage assay confirmed that LEO did not compromise cellular membrane integrity. Across all tested concentrations, the relative viability of L929 cells remained within the range of 94% to 97% when compared with the negative control culture. Additionally, no dose-dependent effect of LEO was observed in any of the assays (Figure 2).

### 2.2. Bird Trial

Production results are summarized in Table 2. The body weight (**BW**) of one-day-old birds was uniform across groups, ranging from 43.11 g to 43.15 g. On day 10 of fattening, chickens in the HE group (received LEO-immobilized hydrogel) had significantly higher BW compared with the control (C) and plain hydrogel (H) groups (*p* < 0.05). No significant differences in feed intake (**FI**) were observed between the groups at any of the rearing time points (*p* > 0.05), with the average FI for the entire fattening period remaining at a similar level (2862.5–2877.8 g/bird). In contrast, the addition of LEO-infused hydrogel capsules significantly improved the feed conversion ratio (**FCR**) during all rearing periods, with overall FCR values ranging from 1.30 (HE) to 1.39 ©. Moreover, significant differences in water intake (**WI**) were found during the first rearing period (days 1–10), when hydrogel capsules were applied (*p* < 0.05). During this time, broilers receiving either hydrogel formulation (H and HE groups) consumed less water than the control group (*p* < 0.05). However, these differences did not persist, and no significant variations in total WI were observed over the entire rearing period (days 1–35), ranging from 6021 mL/bird in the C group to 6035.5 mL/bird in the H group. Broiler survival rates were high and did not significantly differ between the groups, ranging from 98% in the control group to 99% in the H and HE groups (*p* > 0.05).

### 2.3. Effect of Supplementation on Cecal Microbiota

The effects of hydrogel and LEO supplementation on the cecal microbial diversity of the broiler chickens are summarized in Table 3. Supplementation with LEO-infused hydrogel (HE group) resulted in a significant reduction in cecal species richness compared with the control group, as measured by both the Chao1 (*p* = 0023) and Fisher’s alpha (*p* = 0.025) indices. In contrast, neither the hydrogel-only (H) nor the LEO-infused hydrogel (HE) treatment had a significant effect on species evenness, as determined by the Shannon and Simpson indices (*p* > 0.05).

**Figure 3:**
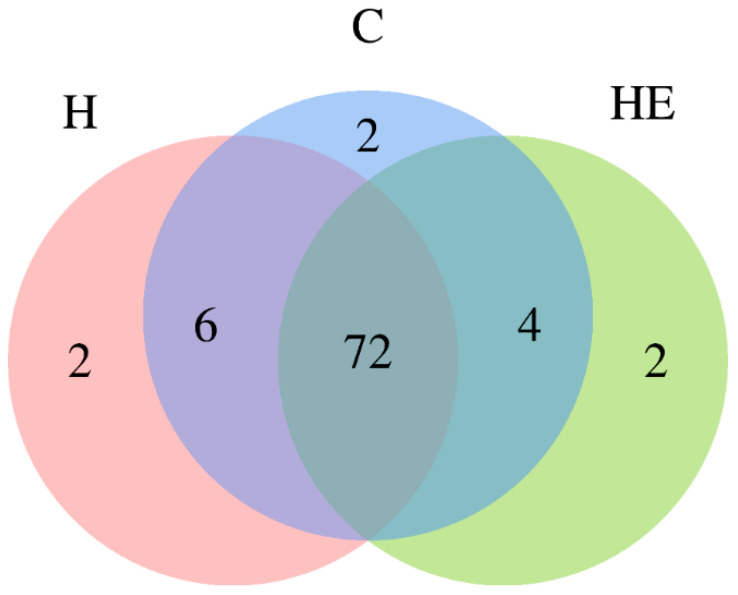
Venn diagram depicting the distribution of bacterial genera in the cecal microbiota digesta between experimental groups. Each ellipse represents a treatment group: control (C), hydrogel (H), and hydrogel supplemented with lavender essential oil (HE). Overlapping regions indicate shared bacterial genera between groups. The analysis included five replications per group (*n* =  5).

**Figure 4:**
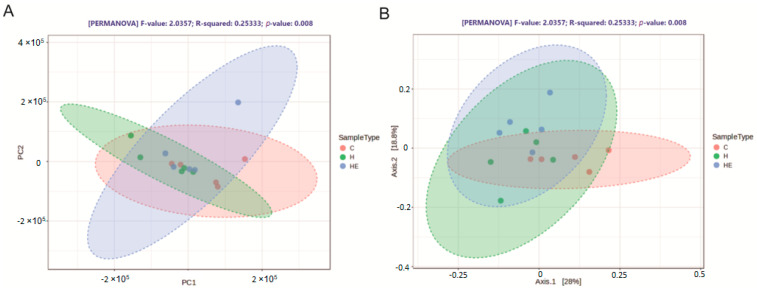
Beta-diversity analysis of cecal microbiota in broiler chickens across treatment groups. (**A**) Principal component analysis (PCA) plot illustrating the variation in cecal microbiota composition control group (C), hydrogel (H), and hydrogel with lavender essential oil (HE) groups. Axes represent the first two principal components. (**B**) Principal coordinate analysis (PCoA) illustrating the variation in cecal microbiota composition between the C, H, and HE groups. Each point of plot represents an individual sample (replicate). Beta-diversity analysis was performed using five replicates per group (*n* =  5).

The Venn diagram revealed that 72 bacterial taxa were shared among all three experimental groups (C, H, HE), representing the core microbiota (Figure 3). Each group also had two unique bacterial taxa. Additionally, six taxa were shared exclusively between the C and H groups, while four taxa were common to the C and HE groups. Notably, no bacterial taxa were found to be unique to both the H and HE groups (Figure 4). Principal component analysis (PCA), based on Bray–Curtis dissimilarity metrics, revealed statistically significant differences in microbiota composition between the groups (*p* = 0.008). Similarly, principal coordinates analysis (PCoA), using the same metrics, indicated clear and statistically significant separation between the groups (*p* = 0.008).

**Figure 5:**
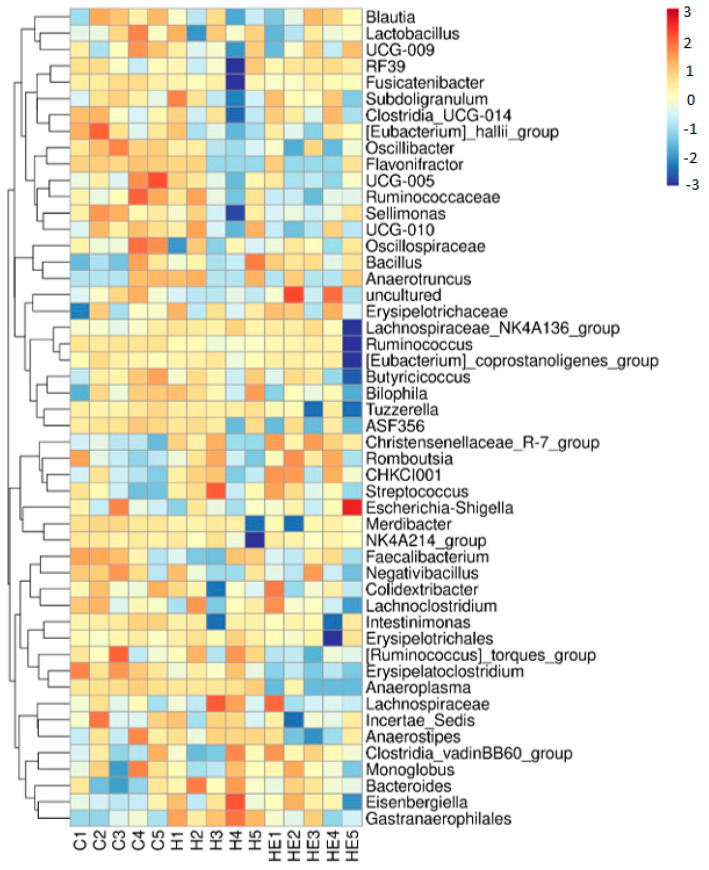
Heatmap analysis of the abundance of cecal microbiota genera. The heatmap displays the relative abundance of the 50 most prevalent bacterial genera (y-axis) across different treatment groups (x-axis): control group (C1–C5), hydrogel group (H1–H5), and hydrogel with lavender essential oil group (HE1–HE5). Each treatment group included five biological replicates (*n* =  5). Abundance values were standardized using Z-score normalization. Color intensity reflects the relative abundance of different bacteria within each sample, with blue indicating lower abundance and red indicating higher abundance.

An overview of the cecal microbiota composition at the genus level is presented in Figure 5. A heatmap of the 50 most abundant genera reveals distinct clustering patterns between the treatment groups. Genera such as *Oscillibacter* and *Flavonifractor*, were more abundant in the C group, while genera, including *UCG-005*, *Ruminococcaceae*, *Sellimonas*, and the *Ruminococcus torques* group, were less abundant in the HE group. In the hydrogel-only (H) group, *Eisenbergiella* and *Gastranaerophilales*, genera showed increased abundance. Additionally, several genera, such as *Tuzzerella*, *Ruminococcus torques group*, and *Anaeroplasma* were partially shared between the C and H groups.

**Figure 6:**
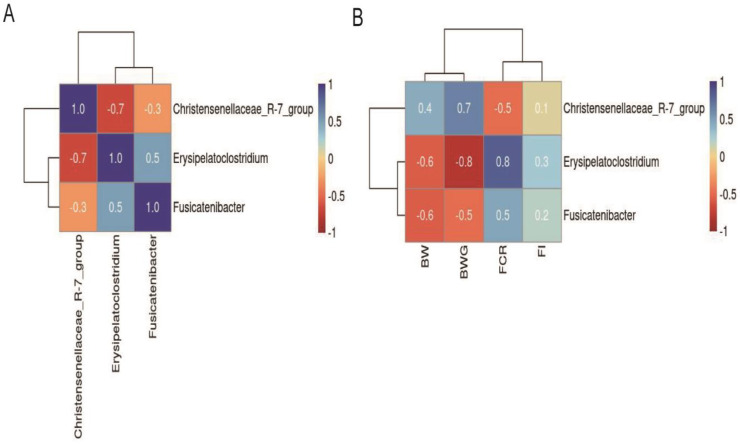
Correlation analysis between cecal microbiota and growth performance in broilers. (**A**) Correlation matrix showing relationships between three dominant microbial genera. (**B**) Correlation matrix between the relative abundances of these genera and growth performance. BW: body weight, BWG: body weight gain, FI: feed intake, FCR: feed conversion ratio. Color gradient indicates correlation strength (blue: positive; red: negative; scale: −1.0 to 1.0). Analyses were performed with five biological replicates per group (*n* = 5).

The effects of hydrogel and hydrogel supplemented with LEO on the bacterial taxonomy in the cecal digesta of broilers are summarized in Table 4. At the phylum level, the abundance of Cyanobacteria was higher in the H group compared with the C and HE groups. At the genus level, the abundance of the genus *Christensenellaceae* R-7 group was higher in the HE group compared with the C group. In contrast, the abundance of the genus *Erysipelatoclostridium* was lower in the HE group compared with the C and H groups. Similarly, *Fusicatenibacter* was less abundant in both the H and HE groups compared with the C group. Correlation analysis between growth performance parameters and dominant genera across groups is presented in Figure 6. A negative correlation was observed between the average abundance of *Christensenellaceae* R-7 group and the genera *Erysipelatoclostridium* and *Fusicatenibacter* (Figure 6A). Additionally, *Erysipelatoclostridium* and *Fusicatenibacter* demonstrated a strong positive correlation with each other (Figure 6A). In terms of production parameters, genus *Christensenellaceae* R-7 group abundance was positively correlated with BW and BWG and negatively correlated with FCR (Figure 6B). Conversely, the average abundance of the genera *Erysipelatoclostridium* and *Fusicatenibacter* was negatively correlated with BW and BWG and positively correlated with FCR (Figure 6B).

## 3. Discussion

The therapeutic potential and preventive applications of EOs and their key constituents, including LEO, are supported by accumulating evidence from in vitro studies and animal models demonstrating their diverse biological activities [12,14,15,16,17,18,19,22,23,24,25]. While traditional applications of LEO are well-documented, modern analytical techniques have enabled more precise characterization of its pharmacological properties and safety profile. Despite the long-term application of LEO in various industries, data regarding its safety profile remain limited. According to current standards, the chemical composition of LEO should comply with either ISO 3515:2002 guidelines or relevant national pharmacopoeial requirements [26]. As defined by the European Pharmacopoeia standards [20], LEO must contain the following chemical components within specified ranges: camphor (<1.2%), linalool (20.0–45.0%), linalyl acetate (25.0–47.0%), terpinen-4-ol (0.1–8.0%), lavandulyl acetate (>0.1%), and α-terpineol (<2.0%). The LEO used in the present study contained predominantly oxidized monoterpenes (linalool and linalyl acetate) and complied with pharmacopeial specifications. In vitro studies using cell lines are a valuable approach for evaluating the cytotoxicity of bioactive compounds, including EOs. While LEO is considered a relatively safe EO, high concentrations may induce cytotoxic effects such as reduced cell viability, membrane damage, oxidative stress, or apoptosis activation [27]. The essential oil used in the current study showed no cytotoxic effects at concentrations up to 0.04%. L929 fibroblast cells maintained high adhesion after 48 h of exposure, demonstrating normal growth and morphology. Cells in both the control cultures (medium + DMSO) and LEO-treated samples showed dense growth, maintained a normal spindle-shaped and elongated morphology, and had no dead cells visible in the field of view. The absence of significant alterations in membrane integrity and morphology confirms the safety of using this EO as a feed additive at concentrations up to 0.04%. The cytocompatibility of LEO was demonstrated in the study of Lupoae et al. [28] in the concentration range of 10–500 μg/mL, where the cell viability (in the NRU assay) varied between 80% and 98% after 24 h of culture. However, higher LEO concentrations (750 and 1000 μg/mL) exerted moderate cytotoxicity, reducing L929 fibroblast viability to 54%. Similarly, after 48 h of exposure, LEO at 750–1000 μg/mL again showed moderate cytotoxic effects, with cell viability ranging from 50% to 70%. In another study, in vitro incubation of periodontal fibroblasts with LEO resulted in an IC_50_ (50% inhibitory concentration) value exceeding >500 μg/mL, indicating relatively low cytotoxicity [29]. Contrasting results from Gavanji et al. [30] indicated no toxic effects of LEO below 12.5 μg/mL. These authors determined a CC_50_ (cytotoxic concentration for 50% of cells) value of 218 μg/mL for *Lavandula officinallis* (MTT assay). Another study using HEK-293 cells (human embryonic kidney 293) also indicated a moderate reduction in cell viability (in MTT assay) by up to approximately 10–20% after 48 h of exposure to LEO concentrations ranging from 23 μg/mL to 187 μg/mL [31]. Variations in cytotoxicity results may be caused by differences in LEO chemical composition, which can be influenced by factors such as plant developmental stage, age, harvested plant parts, seasonal variations, environmental conditions [32,33,34], and also drying methods [35,36].

EOs exert their biological effects through multiple mechanisms, including intestinal acidification, antimicrobial activity, antioxidant properties, anti-inflammatory action, and immunostimulation. These substances influence immune responses and gut microbial balance, thereby stimulating appetite, enhancing digestive processes, and improving nutrient digestibility and absorption [37,38,39]. When properly selected, EOs can also exhibit antidiarrheal effects and promote digestive enzyme production [40,41]. The present experiments conducted showed that dietary supplementation with alginate hydrogel capsules containing immobilized LEO (HE) positively influenced broiler production parameters compared with both the control group (C) and the group receiving alginate hydrogel alone (H). The significantly higher body weight in the HE group, both on days 10 and 35 of the rearing period, indicated the growth-promoting potential of LEO. Interestingly, despite the lack of significant differences in feed intake between experimental groups, the HE group showed reduced FCR, indicating more efficient nutrient utilization. Additionally, broilers receiving hydrogel supplementation, particularly during the early stage of the rearing period, showed decreased WI, likely due to the physicochemical properties of hydrogels. The application of alginate hydrogel with immobilized LEO represents an innovative dietary supplementation strategy combining controlled release capabilities of hydrogels with well-documented health-promoting properties of EOs. While existing literature documents various applications of EOs in poultry nutrition, this specific delivery method remains unexplored [15,18,22,23,42,43,44]. Current research shows inconsistent results regarding dietary EO supplementation, likely due to variations in dosage, administration method (water vs. feed), and differences in bioactive compound composition. Similarly, studies on hydrogel supplementation alone report variable effects on poultry performance and health, particularly during the early rearing stage [45,46,47]. These differences may stem from physiological differences between chicks and adult birds. After hatching, chicks transition from relying on nutrient absorption from the yolk sac to digesting plant-based proteins and carbohydrates provided in feed. The early postnatal period is crucial for the establishment of a balanced gut microbiota and the maturation of the immune system. Therefore, hydrogels present a promising platform for delivering bioactive compounds, such as probiotics and vitamins, and EOs during this important developmental window. For every poultry producer, it is essential that every bird introduced to the farm promptly receive access to drinking water and feed, as their early intake stimulates gastrointestinal tract and digestive system development [48]. Moreover, proper hydration is also vital for optimal nutrient absorption. Hydrogels, which can be produced as cubes or capsules, offer a practical solution in poultry hatcheries. A study by Maman et al. [49] found that day-old chicks exposed to elevated body temperatures (42.6 °C) experienced greater dehydration-related weight loss and reduced organ mass within 12 h post-hatch, ultimately leading to poorer broiler performance. In contrast, no significant performance differences were observed between chicks maintained in control (40.0 °C) and low-temperature (38.1 °C) conditions. This finding suggests that newly hatched chicks are more susceptible to high-temperature stress than low-temperature exposure, highlighting the potential of hydrogels to mitigate transport-related overheating and dehydration. Lowman and Parkhurst [45] demonstrated that the application of a commercial hydrogel preparation significantly reduced body weight loss in emus (*Dromaius novaehollandiae*), while Areaaer et al. [47] reported improved chick growth performance following post-hatch hydrogel application. However, a study by Özlü et al. [50] found no significant benefits associated with hydrogel administration. Despite promising results, current research still lacks comprehensive data on the effects of hydrogels on avian gastrointestinal physiology. It is hypothesized that hydrogel application, by providing rapid water access, may stimulate digestive system development in newly hatched poultry [51].

EOs have been reported to possess significant antibacterial, anti-parasitic, and antifungal activities [52,53,54]. Therefore, EOs could serve as beneficial feed additives that support antimicrobial management in broilers through direct antimicrobial effects or indirect modulation of gut microbiota, potentially reducing the reliance on conventional antibiotics while maintaining animal health and performance. EOs are supported to initiate damage to bacterial cells, facilitating the activity of antibacterial drugs and, consequently, preventing the emergence of increasing resistance to antimicrobial agents. Some studies have reported that supplementation with EOs in the diet has an impact on the gut microbial composition in broilers [52]. It has been demonstrated that the gut microbial composition is associated with the growth performance of broilers [55]. Dietary supplementation of 400 mg/kg thymol- and carvacrol-based EOs increases the cecal species richness in broilers [56]. Supplementation with 100 mg/kg *p*-cymene-based EOs results in a higher cecal species evenness in broilers [57]. In the present study, hydrogel alone did not affect the microbial alpha-diversity in the cecal digesta of broilers. However, dietary supplementation of hydrogel in combination with LEO could reduce the cecal species richness in broilers, indicating that LEOs are an important contributing factor to the composition of the gut microbiota in broilers. The EOs derived from different plant sources exhibit different antibacterial, anti-parasitic, and antifungal properties [53,58]. The difference in microbial alpha-diversity among these studies may be attributed to the distinct composition and concentrations of bioactive compounds of EOs. Previous studies have demonstrated that LEO exhibits antibacterial activities and modulates the ileal bacteria in broilers [15,59]. The cecal species richness was reduced in the HE group, but the growth performance was still better than the C and H groups. It implies that reduced bacterial species by LEO treatment in the cecal microbiota have a beneficial effect on broiler growth, possibly through the targeted reduction of potential pathogens in the gut. Taken together, supplementation with LEO could alter the cecal microbial structure of broilers, and these effects are mainly through its antibacterial activities.

Another study showed that the average abundance of the genus *Christensenellaceae* R-7 group was positively correlated with the growth performance in stressed broilers [60]. Supplementation with blended oil derived from plant and animal sources has been found to increase the abundance of this taxon in the cecal digesta of broilers [61]. The genus *Christensenellaceae* R-7 group has recently been linked to improved gastrointestinal tract development and intestinal health [62,63,64]. In contrast, the genus *Erysipelatoclostridium*, a Gram-positive anaerobic pathogen, has been associated with various diseases in humans, such as osteomyelitis, pseudomembranous colitis, and arthritis [65,66]; it is also more abundant in the feces of obese individuals [67] and has been connected to tibial dyschondroplasia in broilers [68]. Probiotic supplementation has been demonstrated to decrease the abundance of *Erysipelatoclostridium* in broilers challenged with necrotic enteritis [69]. In the current study, dietary supplementation of hydrogel in combination with LEO increased the abundance of the genus *Christensenellaceae* R-7 group while decreasing *Erysipelatoclostridium* in broiler cecal digesta. Moreover, the size of the *Christensenellaceae* R-7 group population was also positively correlated with improved growth performance, suggesting that an increased proportion of this bacterial group may exert beneficial effects on gut health. In contrast, the average abundance of the genus *Erysipelatoclostridium* was negatively correlated with the growth performance of broilers, indicating that adding hydrogel in combination with LEO may reduce the adverse effects on gut function. A negative correlation was also observed between the *Christensenellaceae* R-7 group and *Erysipelatoclostridium*, supporting the idea that this dietary intervention modulates gut microbiota by beneficial bacteria and suppresses pathogens. This microbial shift may help explain why broilers in the HE group performed better than other groups. In another study conducted on laying hens, it showed that EO supplementation did not affect the cecal flora composition and lpha diversity index; it increased the abundance of *Firmicutes*, *Intestinimonas,* and *Megamonas* and decreased *Spirochaetota* and the abundance of *Proteobacteria* [70]. The study of Mokhtari et al. [71] showed that broilers fed 600 mg/kg lavender essence had lower counts of cecal *Lactobacillus* spp. than other groups. *E. coli* and *Coliform* counts were decreased in birds fed 600 mg/kg of lavender; it can be concluded that lavender essence can successfully be used as an antibacterial agent in broiler diets without any detrimental effect on growth performance. In Özbilgin’s study, LEO added to the quail diet was observed to increase the number of *Lactobacillus* spp. colonies. As a result, the authors suggested continuing research on the addition of LEO to poultry rations, as it would have important consequences for poultry production [72].

## 4. Materials and Methods

### 4.1. Lavender Essential Oil and Alginate Hydrogels

The commercial LEO used in this study was isolated from the lavender (*Lavandula angustifolia*) flowers (Avicenna Oil, Wrocław, Poland). Its chemical composition was determined using chromatographic analysis performed with a 6890 N gas chromatograph (Agilent Technologies, Palo Alto, CA, USA) equipped with a 5973 Network mass detector and a 7683 Series automatic sample injector. Chromatographic conditions were optimized for analyte separation using an HP-5MSI capillary column (30 m × 0.25 mm). The thickness of the stationary phase base was 0.25 µm. The injector temperature was set to 250 °C, ion source to 230 °C, and quadrupole to 150 °C. Samples (3 µL) were injected using split mode (1:10) and analyzed in scan mode (*m*/*z* 40–500). The column temperature was ramped from 60 °C (3 min hold) to 300 °C at 10 °C/min and held for 13 min. Compounds were identified by matching mass spectra and retention indices with literature data (Supplementary Materials) [21]. Retention indices were determined using a C7–C30 n-alkane standard (Supelco, Bellefonte, PA, USA) analyzed under the same conditions. Relative percentages were calculated based on peak area ratios to total ion current. All analyses were performed in triplicate. LEO was immobilized in an alginate hydrogel at 0.4 mL/L water using Tween 20 emulsifier (Sigma-Aldrich, St. Louis, MO, USA), applied in a 1:1 ratio with the LEO. The extrusion method was used to produce hydrogel capsules. Capsules were produced using the extrusion method by APRS S.A. (Nielbark, Kurzetnik, Poland) in collaboration with the West Pomeranian University of Technology in Szczecin. The method was based on the principle of changing the voltage of a charged liquid, which decreased with increasing electrostatic potential. This potential was generated between an electrode immersed in an electrolyte solution (300 mM CaCl_2_, Biomus, Lublin, Poland) and a syringe needle. The polymer solution in the syringe, consisting of 6% sodium alginate mixed with LEO and emulsifier (1:1 *v*/*v* ratio), was pumped through the needle. As droplets formed at the needle tip, gravity and the electrostatic field (maintained by a magnetic stirrer) guided them into the gelling solution (300 mM CaCl_2_, Biomus, Lublin, Poland), resulting in the formation of a capsule. After formation, the capsules were filtered and rinsed with CaCl_2_ solution (10 mM). The final product was stored in this solution at room temperature until the start of the experiment. The encapsulation efficiency *w*/*w*% (EE%) and loading capacity *w*/*w*% (LC%) of LEO were 80.45% and 53.60%, respectively. The *EE%* and *LC%* of LEO were calculated according to Formulas (1) and (2) as follows:

(1) $$\mathrm{Encapsulation}\;\mathrm{efficiency}\;(\%)=\left(\frac{Total\;amount\;of\;loaded\;LEO\;(\%\;in\;hydrgel)}{Initial\;amount\;of\;LEO\;(\%\;in\;intial\;solution)}\right)\times \;100$$

(2) $$\mathrm{Loading}\;\mathrm{capacity}\;(\%)=\left(\frac{Total\;amount\;of\;loaded\;LEO\;(amount\;in\;oil)}{Weight\;of\;hydrogel\;after\;loading}\right)\;\times \;100$$

### 4.2. Cytotoxicity of Lavender Essential Oil

*Cell culture and experimental conditions.* The biological activity of LEO was analyzed using a normal mouse L929 fibroblast cell line (L cell, L-929, derivative of strain L, ATCC^®^ CCL-1™). Cells were seeded into 96-well plates (TPP, Techno Plastic Products AG, Trasadingen, Switzerland) at a density of 3.5 × 10^3^ per well (for CCK-8, LDH, and NRU assays) and into 12-well plates at 4 × 10^4^ per well (for morphological analysis) and were maintained under standard culture conditions (37 °C, 5% CO_2_, 95% relative humidity) prior to experimental treatment. The complete DMEM culture medium (Dulbecco’s Modified Eagle Medium, High Glucose, Capricorn Scientific, GmbH, Ebsdorfergrund, Germany) was supplemented with 10% fetal bovine serum (Sigma-Aldrich, St. Louis, MO, USA), 2 mM L-glutamine (Corning Inc., Corning, NY, USA), 50 IU/m penicillin, 50 µg/mL streptomycin (Sigma-Aldrich, St. Louis, MO, USA), and amphotericin B (2.5 μg/mL; Sigma-Aldrich, St. Louis, MO, USA). After 24 h, six LEO concentrations—(0.001%, 0.002%, 0.004%, 0.01%, 0.02%, and 0.04%)—were prepared in DMEM culture medium containing 0.1% final DMSO and added to the cultures. Three control cultures were also prepared—negative control (DMEM without DMSO or LEO), DMSO vehicle control (0.1% DMSO), and positive control (6 μM 10-hydroxycamptothecin). Cells were maintained in experimental conditions for 48 h before analysis.

*Cellular morphology analysis.* The morphology of the L929 cells was evaluated after 48 h using phase contrast microscopy. Observations were conducted with a Nikon TS-100 inverted phase contrast microscope equipped with a CFI Achromat DL 10× objective lens (NA 0.25, WD 6.2 mm, Ph1) and a Nikon DS-Fi1 5-megapixel camera. Image acquisition was performed at 100x magnification using NIS Elements F software (v4.00.06, Nikon, Melville, NY, USA).

*CCK-8 assay—determination of cell viability.* The relative cell viability of L929 cells after 48 h exposure to the tested factor was determined using the Cell Counting Kit-8 (Vazyme, Red Maple Hi-tech Industry Park, Nanjing, China). The CCK-8 assay is based on the conversion of WST-8 (2-(2-methoxy-4-nitrophenyl)-3-(4-nitrophenyl)-5-(2,4-disulfophenyl)-2Htetrazolium, monosodium salt) into colored formazan by cellular dehydrogenases of viable cells. After treatment, 10 µL of CCK-8 solution was added to each well, and the plates were incubated for 2 h at 37 °C. The absorbance was recorded at 450 nm with a reference wavelength of 650 nm using a Sunrise Absorbance Reader (Sunrise, Tecan, Männedorf, Switzerland), as was described elsewhere [73]. All the analyses were performed in three independent experiments.

The LEO biological activity on relative cell viability was calculated using the following Equation (3):

(3) $$\mathrm{Relative}\;\mathrm{viability}\;\mathrm{from}\;\mathrm{CCK}\text{-}8\;\mathrm{assay}\;(\%)=\left(\frac{{\mathrm{sample}\;A}_{450–650\;\mathrm{nm}}}{{\mathrm{positive}\;\mathrm{control}\;A}_{450–650\;\mathrm{nm}}}\right)\times \;100$$

where A is the absorbance at 450–650 nm.

*Neutral red uptake assay analysis.* The effect of the LEO on L929 cells was also monitored using the neutral red uptake (NRU) assay (In Vitro Toxicology Assay Kit, Neutral Red based, Sigma-Aldrich, St. Louis, MO, USA). This assay is based on the active uptake of neutral red (NR) dye by viable cells and its accumulation in lysosomes. After the 48 h exposition of L929 cells to LEO, they were washed with phosphate-buffered saline (1xDPBS) (Corning Inc., Corning, NY, USA) and incubated with whole culture medium containing 10% of NR for 3 h under standard conditions. After incubation, the cells were washed again with DPBS, and Neutral Red Assay Solubilization Solution (Sigma-Aldrich, St. Louis, MO, USA) was added to cells. The absorbance was measured at 540 nm with background correction at 690 nm using a Tecan Sunrise microplate reader (Sunrise, Tecan, Männedorf, Switzerland). All the analyses were conducted in three independent experiments [74].

The relative cell viability was calculated using the following Equation (4):

(4) $$\mathrm{Neutral}\;\mathrm{red}\;\mathrm{uptake}\;\mathrm{assay}\;(\%)=\left(\frac{{\mathrm{sample}\;A}_{540–690\;\mathrm{nm}}}{{\mathrm{positive}\;\mathrm{control}\;A}_{540–690\;\mathrm{nm}}}\right)\times \;100$$

where A is the absorbance at 450–650 nm.

*Lactate dehydrogenase (LDH) leakage assay.* The membrane integrity of L929 cells following 48 h cell exposure to LEO was evaluated using the LDH CytoTox 96^®^ Non-Radioactive Cytotoxicity Assay (Promega, Madison, WI, USA). Lactate dehydrogenase (LDH) is a cytosolic enzyme that is released into the culture medium due to cellular membrane damage. The level of LDH release upon cell lysis was determined by measuring absorbance at 490 nm using a Tecan Sunrise microplate reader (Sunrise, Tecan, Männedorf, Switzerland). All the analyses were conducted in three independent experiments [74].

The relative cell viability was calculated using Equation (5) described by Verrax and Calderon (2011) [75]:

(5) $$\%\mathrm{LDH}\;\mathrm{released}=100\%\;-\;(\frac{A490\;\mathrm{nm}\;\mathrm{of}\;\mathrm{treated}\;\mathrm{and}\;\mathrm{untreated}\;\mathrm{cells}-A490\;\mathrm{nm}\;\mathrm{of}\;\mathrm{control}}{A490\;\mathrm{nm}\;\mathrm{of}\;\mathrm{maximum}\;\mathrm{of}\;\mathrm{untreated}\;\mathrm{cells}-A490\;\mathrm{nm}\;\mathrm{of}\;\mathrm{control}}\;\times \;100)$$

where A is the absorbance at 490 nm.

### 4.3. Bird Trial

The experiment was conducted on a commercial poultry farm with Veterinary Identification Number 32044946 (Żabówko, Nowogard, Poland) during a standard production cycle using unsexed Ross 308 broiler chickens. The birds were purchased from a commercial hatchery (Park Drobiarski Sp. z o.o., Śmiłowo, Kaczory, Poland). Throughout the trial, birds remained under veterinary supervision by the District Veterinary Officer in Goleniów for health monitoring. The chickens were housed in a poultry house in designated pens. Group allocation was performed by farm staff who were unaware of the experimental design, ensuring unbiased randomization. One-day-old chicks were randomly assigned to three experimental groups of 100 individuals each (5 replicates, 20 birds per group). The control group (**C**) did not receive any hydrogel supplementation throughout the rearing period. Groups **H** and **HE** were provided with hydrogel capsules from days 1–10 of rearing, administered on chick paper alongside standard feed for voluntary consumption. Chick paper is an industry-standard material designed to optimize early feeding behavior in poultry. This high-rustling paper was placed beneath watering lines, where its distinctive sound when stepped on attracted chicks to the feed and water area. The H group received hydrogel alginate capsules without essential oil, while the HE group was supplemented with hydrogel capsules containing immobilized LEO (0.4 mL/L). Capsules were administered at a rate of 2 kg applied twice daily onto chick paper, with all capsules being fully consumed. The total number of capsules per pen was calculated based on the number of chicks and the target dosage per bird, with a 10% excess to account for potential wastage. Capsules were distributed at four equidistant points within each pen to ensure uniform access. Fresh capsules were applied daily at 8:00 and 18:00 for 10 consecutive days. Pens were monitored every 2 h during daylight to observe consumption patterns and chick behavior.

The chickens had unrestricted access to water drinkers during the entire experiment. All birds were housed in the same poultry house for 35 days on wheat straw bedding at a stocking density of 14 birds/m^2^. Broilers were kept under standardized environmental conditions in accordance with the Ross 308 guidelines [76], with parameters monitored daily. The temperature was maintained at 32–33 °C during days 1–2, then gradually reduced according to schedule: 30 °C (day 3), 28–30 °C (days 4–7), 26–28 °C (days 8–14), 24–26 °C (days 15–21), and approximately 22 °C (days 22–41). Relative humidity was progressively increased from 50% at the start to 70% by the end of the trial. Birds received ad libitum commercial feed (Polskie Zakłady Zbożowe Sp. z o.o., Wałcz, Poland) consisting of starter (days 1–10), grower I (days 11–20), grower II (days 21–30), and finisher (days 31–35) formulations. The complete feed composition and nutritional values are provided in Supplementary Table S1. The study complied with all Polish regulations regarding experimental animals, strictly following national and institutional guidelines for ethical animal use. All procedures related to animal handling, euthanasia, experimental protocols, and biosecurity were carried out under strict supervision in a specialized poultry facility designed to ensure animal welfare. The research maintained rigorous biosecurity measures and guaranteed that no animals experienced pain, suffering, distress, or lasting harm. Throughout the experiment, body weight (BW; days 1, 7, 14, 21, 28, and 35), feed intake (FI), water intake (WI), and survival rate were recorded. On day 35, two birds per replicate were randomly selected for intestinal scoring. Euthanasia was humanely performed via decapitation by certified personnel. Following euthanasia, necropsies were performed, and the gastrointestinal tracts were dissected into anatomical sections. The ceca were aseptically isolated, the samples were coded, flash-frozen, and stored at −82 °C until analyses.

### 4.4. 16S rRNA Sequencing and Analysis

Microbial DNA was extracted from cecal digesta using the ZymoBIOMICS DNA Miniprep Kit (Zymo Research, Irvine, CA, USA). Agarose gel electrophoresis was performed to assess the concentration and purity of the extracted DNA. The V3–V4 hypervariable region of the bacterial 16S rRNA gene was amplified using 338F and 806R primers. DNA amplicons generated from individual samples were excised from agarose gel electrophoresis and then purified using a QIAquick Gel Extraction kit (QIAGEN, Germantown, MD, USA). Paired-end sequencing (2 × 300 bp) was carried out using Illumina MiSeq 300PE platform (Illumina, San Diego, CA, USA). The raw sequencing data were subjected to quality control using FastQC software (version 0.12.1). Adapter and primer sequences were trimmed using Trimmomatic (version 0.39) and Cutadapt software (version 3.5), and low-quality reads (<Q20) were removed. Quality-filtered reads were subsequently processed using DADA2 (v1.26.0) for denoising, error correction, chimera removal, read merging, and dereplication. Representative sequences and amplicon sequence variant (ASV) tables were generated for each sample. Taxonomic annotation of ASVs was performed using the QIIME2 feature-classifier plugin (v2024.10.0) against the SILVA rRNA gene database (version 138) at 99% sequence similarity. Differences in microbial community composition and relative abundance were analyzed using QIIME2 (version 2024.10.0). Alpha-diversity indices (Chao1, Fisher alpha, Shannon, and Simpson) and beta-diversity comparisons (principal component analysis and principal coordinate analysis) were conducted using the MicrobiomeAnalyst 2.0 online platform. The relative abundance of bacterial taxa was visualized by using the R package heatmap (version 1.0.12).

### 4.5. Statistical Analysis

All statistical analyses were performed using SAS (version 9.4, 2012; SAS Institute, Cary, NC, USA). Individual pens were considered replicates, with each replicate defined as an experimental unit. The data were tested for normality by using the Shapiro–Wilk test. Differences between groups were evaluated using the Kruskal–Wallis test followed by Dunn’s pairwise comparisons. Principal component analysis (PCA) and principal coordinate analysis (PCoA) based on Bray–Curtis dissimilarity metrics were performed to determine microbial composition similarities. Pearson’s correlation analysis was used to assess relationships between growth performance and the relative abundance of bacterial taxa. Statistical significance was set at *p* ≤ 0.05.

## 5. Conclusions

The present results demonstrate the potential of supplementing alginate hydrogels with immobilized LEO during the initial feeding phase as a natural growth promoter in broiler chickens. This approach supports growth primarily through beneficial modulation of the gut microbiota and improved feed utilization. Immobilization of LEO in alginate hydrogel enables a controlled release of the active compound in the gastrointestinal tract, minimizing losses, enhancing bioavailability, and improving functional efficacy. This innovative strategy aligns with efforts to reduce antibiotic use and promote natural alternatives in poultry production. However, the observed reduction in microbial biodiversity raises concerns regarding potential impacts on immune function and gastrointestinal health, requiring further research on the long-term effects of these additives, including their influence on meat quality and animal welfare. Future research should also focus on optimizing the dosage, timing, and delivery methods, as well as elucidating the underlying molecular mechanisms responsible for the observed biological effects. The present findings have important implications for the poultry industry by offering a natural, environmentally friendly alternative to conventional antimicrobial growth promoters, contributing to the development of more sustainable and responsible livestock production systems.

## Figures and Tables

**Table 1:** Chemical composition of essential oil from lavender (*Lavandula angustifolia*) essential oil.

Compound	RI ^1^ (RI Ref) ^2^	Percentage (Relative Peak Area) ± SD ^3^
α-Pinene	934 (935)	0.81 ± 0.09
Camphene	946 (950)	0.45 ± 0.05
β-Pinene	976 (977)	0.65 ± 0.08
β-Myrcene	989 (989)	0.58 ± 0.01
Hexyl acetate	1008 (1010)	0.61 ± 0.06
δ-3-Carene	1010 (1011)	0.20 ± 0.01
*p*-Cymene	1022 (1024)	0.85 ± 0.05
Limonene	1028 (1029)	0.15 ± 0.06
Eucalyptol	1031 (1031)	1.75 ± 0.08
(Z)-β-Ocimene	1037 (1038)	2.70 ± 0.21
(E)-β-Ocimene	1047 (1048)	0.10 ± 0.01
Linalool oxide	1074 (1075)	1.14 ± 0.01
Linalool	1101 (1099)	31.55 ± 0.91
Camphor	1145 (1144)	0.35 ± 0.02
Borneol	1165 (1166)	1.85 ± 0.10
4-Terpineol	1177 (1177)	2.05 ± 0.05
*p*-Cymen-8-ol	1183 (1184)	0.50 ± 0.01
α-Terpineol	1191 (1191)	0.35 ± 0.02
Linalyl acetate	1256 (1256)	41.20 ± 1.01
Bornyl acetate	1280 (1282)	0.10 ± 0.01
Lavandulyl acetate	1289 (1291)	2.22 ± 0.02
Cuminic alcohol	1293 (1295)	0.10 ± 0.01
Neryl acetate	1362 (1364)	0.80 ± 0.09
Geranyl acetate	1379 (1380)	0.19 ± 0.01
α-Copaene	1381 (1381)	0.14 ± 0.04
β-Caryophyllene	1419 (1420)	2.59 ± 0.10
(E)-β-Farnesene	1455 (1456)	0.15 ± 0.01
Caryophyllene oxide	1577 (1577)	2.15 ± 0.11
Epi-bicyclosesquiphellandrene	1641 (1642)	0.81 ± 0.15

^1^ Linear retention index determined experimentally in relation to n-alkanes (C_7_–C_30_) on a HP5-MSI column; ^2^ Reference linear retention index from the literature [21]. ^3^ Standard deviation (*n* = 3).

**Table 2:** Effect of alginate hydrogel supplementation on broiler growth.

Day ^3^	Group ^1^			SEM ^4^	*p*-Value ^2^		
	C	H	HE		C vs. H	H vs. HE	HE vs. C
BW (g/bird)							
1	43.31	43.15	43.26	0.07	1.000	1.000	1.000
10	279.10	275.55	293.12	5.14	0.55	0.023	0.001
35	2119.63	2121.20	2250.51	14.61	0.67	0.041	0.001
BWG (g/bird)							
1–10	235.79	232.40	250.0	4.15	0.662	0.031	0.038
11–35	1840.53	1845.65	1957.39	15.8	0.891	0.036	0.021
1–35	2076.32	2078.05	2207.25	21.51	0.910	0.001	0.001
FI (g/bird)							
1–10	264.30	262.9	265.50	1.89	0.922	0.988	0.942
11–35	2613.5	2599.60	2609.20	9.21	0.923	0.989	0.983
1–35	2877.8	2862.5	2874.7	13.41	0.899	0.933	0.934
FCR (g/g)							
1–10	1.12	1.13	1.06	0.03	1.000	0.047	0.046
11–35	1.42	1.41	1.33	0.04	1.000	0.021	0.020
1–35	1.39	1.38	1.30	0.05	1.000	0.034	0.031
WI (mL/bird)							
1–10	684.70	665.80	660.43	7.56	0.049	0.56	0.039
11–35	5329.3	5369.7	5383.38	18.55	0.892	0.864	0.877
1–35	6021.0	6035.5	6033.81	20.87	0.874	0.799	0.768

^1^ C: Control; H: Hydrogel; HE: Hydrogel + lavender essential oil. ^2^ Kruskal-Wallis test, Statistical significance is set at *p* ≤ 0.05. ^3^ BW: body weight; BWG: body weight gain; FI: feed intake; FCR: feed conversion ratio; WI: water intake. ^4^ SEM: Standard error of measurement.

**Table 3:** Alpha diversity of cecal microbiota.

					*p*-Value ^2^			Effect Size
Item	C ^1^	H	HE	SEM ^3^	C vs. H	H vs. HE	HE vs. C	
Chao1	62.0	60.0	55.8	0.93	0.856	0.170	0.023	0.43
Fisher alpha	8.0	7.9	7.3	0.11	0.947	0.178	0.025	0.39
Shannon	3.0	2.8	2.8	0.05	0.178	0.947	0.116	0.24
Simpson	0.9	0.9	0.9	0.01	0.363	0.861	0.363	0.06

^1^ C: Control; H: Hydrogel; HE: Hydrogel + lavender essential oil. ^2^ Kruskal–Wallis test, Statistical significance is set at *p* ≤ 0.05. ^3^ SEM: Standard error of measurement.

**Table 4:** Relative abundance of the bacterial taxa in the cecal digesta of broiler chickens.

	Group ^1^				*p*-Value ^2^			Effect Size
Item	C	H	HE	SEM ^3^	C vs. H	H vs. HE	HE vs. C	
Phylum								
Firmcutes	75.7	66.5	67.9	2.28	0.363	0.947	0.615	−0.01
Bacteroidota	17.9	28.4	22.8	2.01	0.363	0.363	0.363	0.11
Proteobacteria	4.3	2.3	7.8	1.81	0.745	0.484	0.861	−0.05
Desulfobacterota	1.8	1.9	1.1	0.20	0.947	0.178	0.363	0.13
Cyanobacteria	0.3	0.8	0.4	0.08	0.025	0.043	0.484	0.61
Genus								
*Bacteroides*	17.9	28.4	22.8	2.01	0.363	0.363	0.363	0.11
*Faecalibacterium*	15.6	12.1	11.8	0.98	0.60	0.994	0.363	0.08
*RF39*	7.1	5.8	8.2	0.60	0.861	0.260	0.615	0.04
*Lactobacillus*	7.4	7.1	4.8	0.77	0.947	0.484	0.484	−0.01
*Clostridia_vadinBB60_group*	4.8	4.8	5.9	0.46	0.947	0.484	0.484	−0.01
*Lachnospiraceae*	4.3	5.9	4.7	0.65	0.947	0.484	0.615	−0.05
*Escherichia-Shigella*	4.3	2.3	7.8	1.81	0.745	0.484	0.861	−0.01
*Clostridia_UCG-014*	4.9	3.9	4.6	0.33	0.363	0.615	0.947	0.23
*Blautia*	3.2	1.6	2.4	0.36	0.260	0.484	0.615	0.10
*CHKCI001*	1.0	2.1	4.0	0.58	0.745	0.484	0.178	0.12
*Christensenellaceae_R-7_group*	0.9	2.4	3.8	0.47	0.615	0.363	0.025	0.38
*Butyricicoccus*	2.7	2.3	1.6	0.28	0.745	0.484	0.484	0.01
*Bilophila*	1.6	1.8	1.0	0.18	0.861	0.116	0.363	0.18
*UCG-005*	1.8	1.2	1.0	0.19	0.745	0.260	0.615	0.06
*Ruminococcus_torques_group*	1.4	1.5	0.7	0.18	0.861	0.178	0.072	0.29
*Erysipelatoclostridium*	1.8	1.2	0.7	0.14	0.116	0.025	0.025	0.76
*Fusicatenibacter*	2.3	0.6	0.5	0.30	0.025	0.861	0.025	0.63
*Incertae_Sedis*	1.0	0.9	0.7	0.08	0.947	0.615	0.861	−0.11
*Bacillus*	0.7	0.9	0.9	0.18	0.745	0.484	0.615	−0.03

^1^ C: Control; H: Hydrogel; HE: Hydrogel with lavender essential oil. ^2^ Kruskal–Wallis test and Dunn’s pairwise test, Statistical significance is set at *p* ≤ 0.05. ^3^ SEM: Standard error of measurement.

## Data Availability

All relevant data are included in the Supplementary Materials.

## Supplementary Materials

The following supporting information can be downloaded at: https://www.mdpi.com/article/10.3390/molecules30142931/s1, Table S1. Ingredient and nutrient composition of the basal diets; Figure S1: Total Ion Chromatogram of *Lavandula angustifolia* essential oil obtained by the GC-MS method; Figure S2: Mass spectrum of linalool present in *Lavandula angustifolia* essential oil, compared with the linalool standard mass spectrum from the NIST 02 library; Figure S3: Mass spectrum of linalool acetate present in *Lavandula angustifolia* essential oil, compared with the linalool acetate standard mass spectrum from the NIST 02 library; Figure S4: Mass spectrum of 4-terpineol present in *Lavandula angustifolia* essential oil, compared with the 4-terpineol standard mass spectrum from the NIST 02 library.

## Abbreviations

The following abbreviations are used in this manuscript:

BW	Body weight
BWG	Body weight gain
DMSO	Dimethylsulfoxide
Eos	Essential oils
FI	Feed Intake
FCR	Feed Conversion Ratio
LEO	Lavender essential oil
WI	Water Intake
