# Supplementary material for: Chemical Composition, Cytotoxicity, and Encapsulation of Lavender Essential Oil (Lavandula angustifolia) in Alginate Hydrogel—Application and Therapeutic Effect on Animal Model
Source: Molecules. 2025 Jul 11;30(14):2931. doi: 10.3390/molecules30142931 (PMC12299666; doi:10.3390/molecules30142931)
Supplement: Supplementary file 1 [file molecules-30-02931-s001.zip › molecules-3714058-supplementary.pdf]

Supplementary material

PART I - Ingredient of diets

Supplementary Table S1. Ingredient and nutrient composition of the basel diets (%)

Item	Starter (0–10 d)	Grower I (11–20 d)	Grower II (21–30 d)	Finisher (31–35 d)
Ingredient				
Wheat, 11.6%	37.00	38.00	33.00	40.00
Soybean meal, 46%	29.05	24.94	27.16	24.0
Maize, 8%	25.93	27.39	28.74	25.32
Soy oil	2.68	2.78	3.79	3.66
Canola meal, 32.5%	-	2.50	2.50	3.01
Potato protein, 73%	1.50	1.0	0.50	-
Limestone	1.27	0.85	0.78	0.67
Monocalcium phosphate	0.92	0.43	0.30	0.11
Vitamin and mineral premix[1]	0.53	0.53	0.60	0.59
Poultry fat	-	0.50	1.50	1.50
L-Lys-HCl	0.39	0.40	0.41	0.43
DL-Met	0.26	0.17	0.18	0.17
Salt	0.24	0.27	0.28	0.28
$NaHCO_3$	0.14	0.13	0.15	0.14
Thr	0.07	0.06	0.07	0.07
Choline chloride	-	0.03	0.02	0.03
Phytase premix[2]	0.02	0.02	0.02	0.02
Calculated analysis				
ME (kcal/kg)	2800.0	2865.0	2984.1	3020.0
Lys	1.26	1.16	1.15	1.07
Met	0.56	0.46	0.45	0.44
Ca	0.86	0.67	0.71	0.65
P	0.56	0.47	0.42	0.39
Na	0.15	0.15	0.15	0.15
Analyzed nutrient composition[3]				
CP	21.50	20.00	19.50	18.50
Crude fiber	2.60	2.50	2.60	3.00
Crude fat	5.80	6.40	6.60	7.00
Crude ash	5.40	4.70	4.20	4.00

[1]Vitamin-mineral premix contained the following per kilogram of diet: vitamin A, 9,000 IU; vitamin D3, 4,000 IU; vitamin E, 60 mg; vitamin B1, 3 mg; vitamin B2, 10 mg; vitamin B6, 3 mg; vitamin B12, 15 μg;

nicotinic acid, 60 mg; pantothenic acid, 14.7 mg; folic acid, 1.5 mg; iron, 59.85 mg; copper, 15 mg; cobalt, 1.0 mg; zinc, 100 mg; iodine, 1.5 mg; selenium, 0.225 mg, antioxidant (BHA), 12.0 mg.
[2]Phytase premix was prepared by dilution with calcium carbonate to contain 1,500 FTU (phytase units)/g (Optiphos, Huvepharma AD, Sofia, Bulgaria)
[3]Based on a DM content of 87.5%

PART II - GC-MS data of *Lavandula angustifolia* essential oil

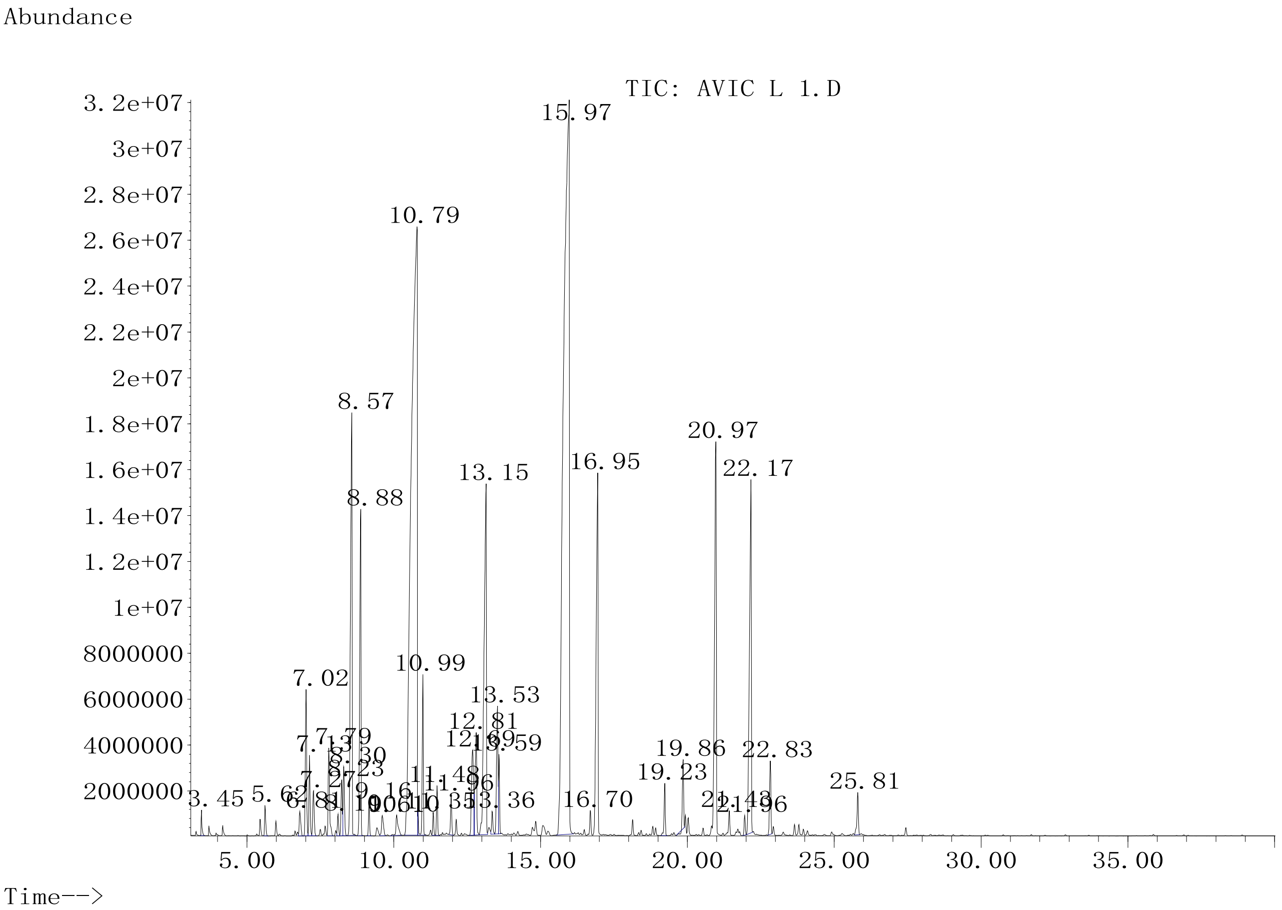

Figure S1. Total Ion Chromatogram of *Lavandula angustifolia* essential oil obtained by GC-MS method.

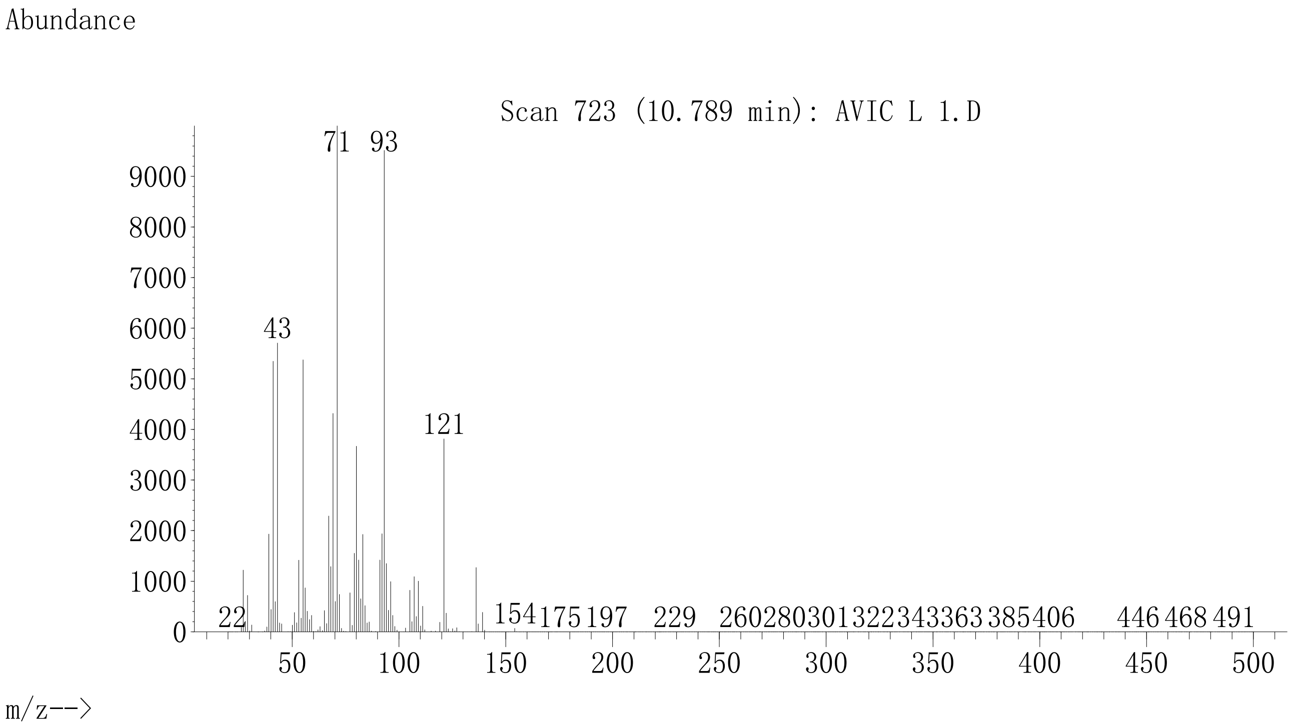

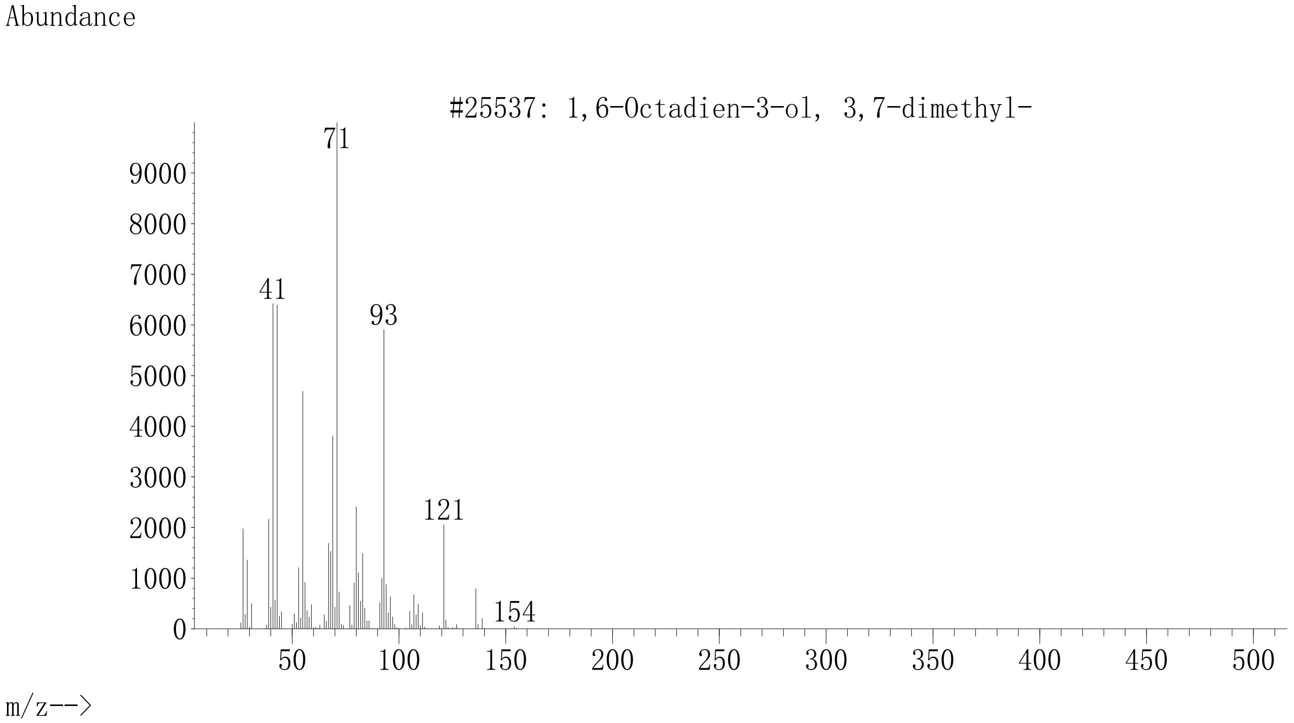

Figure S2. Mass spectrum of linalool present in *Lavandula angustifolia* essential oil, compared with linalool standard mass spectrum from NIST 02 library.

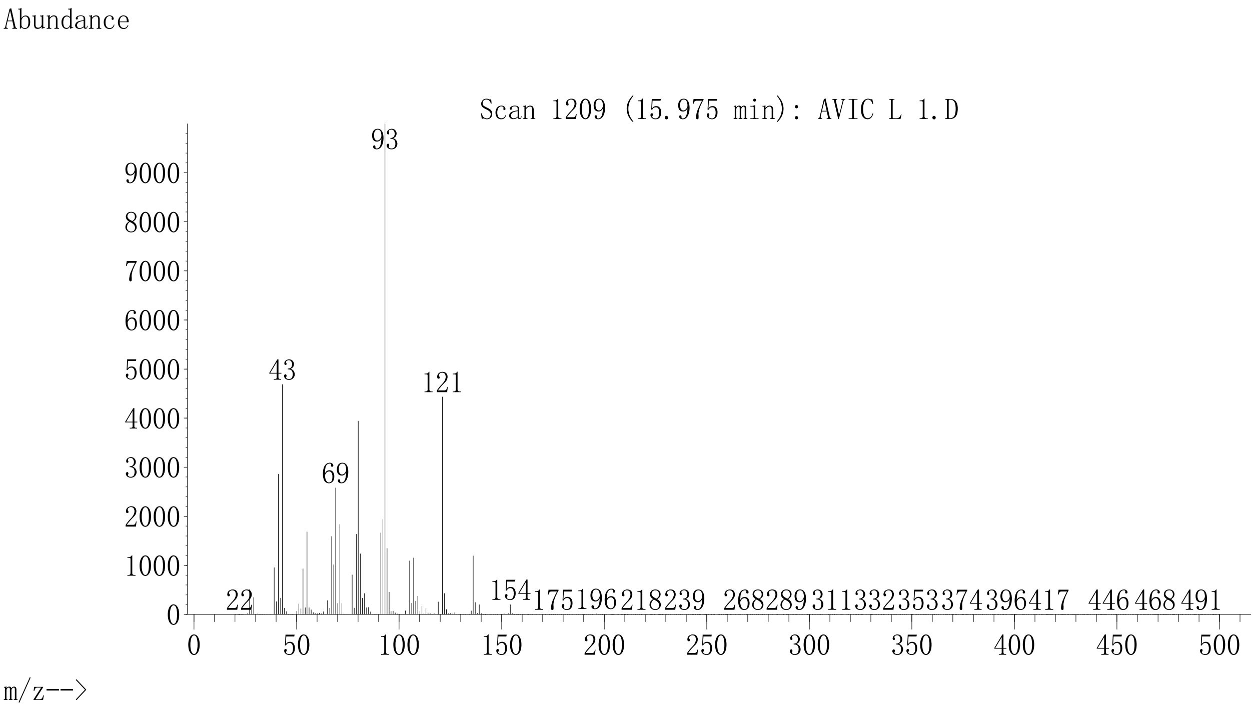

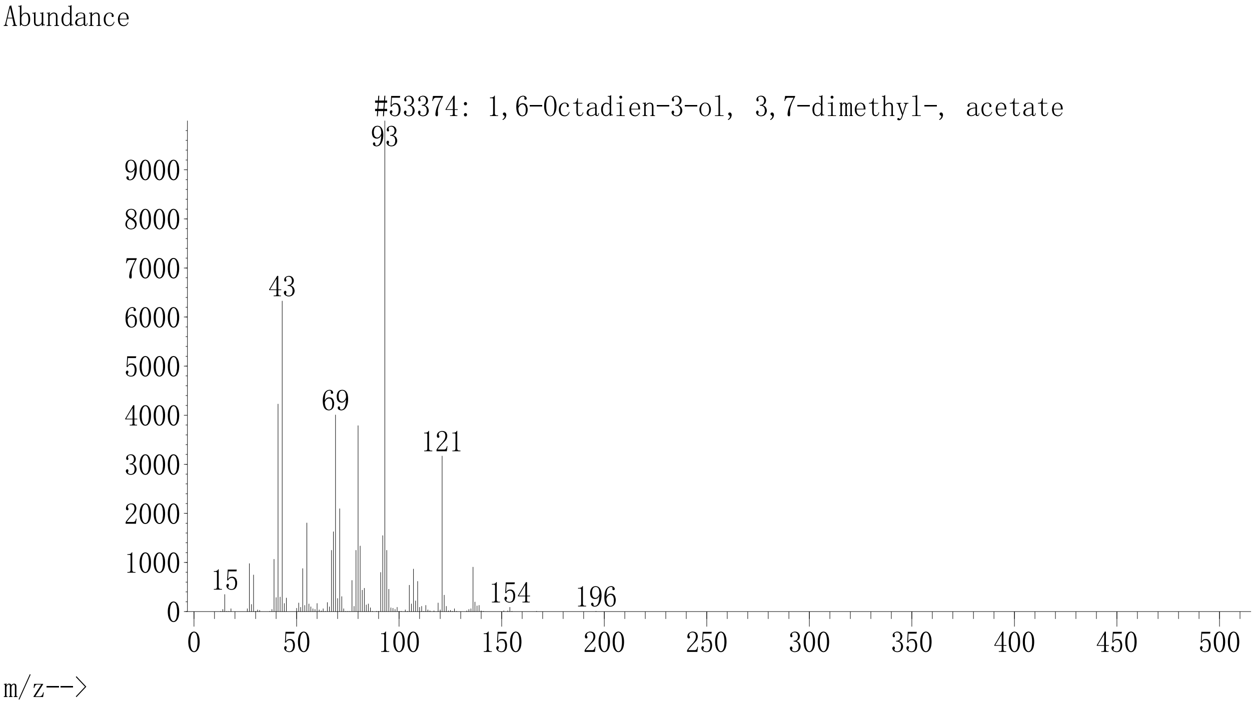

Figure S3. Mass spectrum of linalool acetate present in *Lavandula angustifolia* essential oil, compared with linalool acetate standard mass spectrum from NIST 02 library.

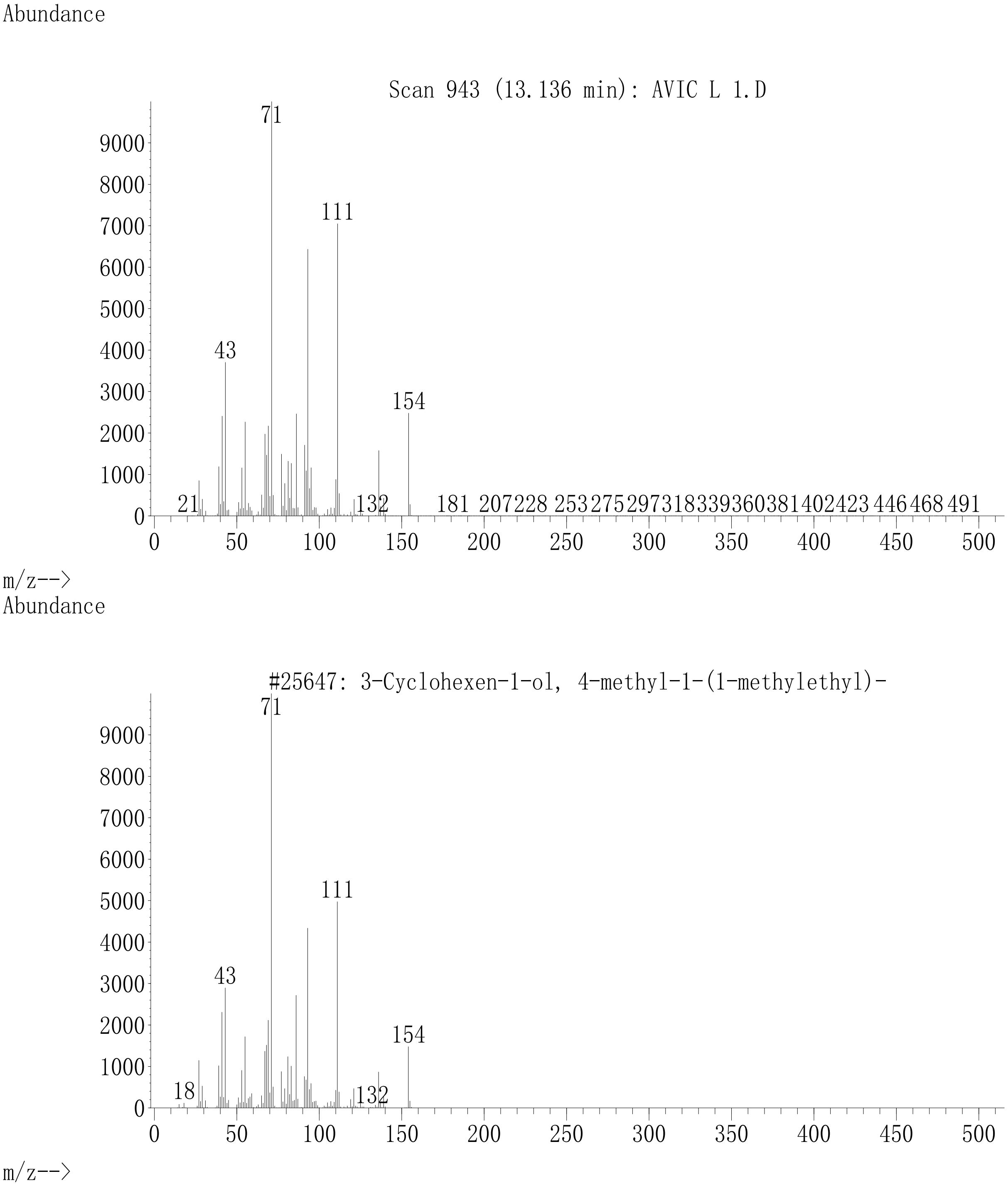

Figure S4. Mass spectrum of 4-terpineol present in *Lavandula angustifolia* essential oil, compared with 4-terpineol standard mass spectrum from NIST 02 library
